# INTRINSIC CAPACITY TRANSITION AND ITS ASSOCIATION WITH INCIDENT DISABILITY: FINDINGS FROM THE KFACS

**DOI:** 10.1093/geroni/igae098.4009

**Published:** 2024-12-31

**Authors:** Hyunjin Cho, Hyung Eun Shin, Heeeun Jung, Daehyun Lee, Jae Young Jang, Nahyun Lim, Chang Won Won, Miji Kim

**Affiliations:** Kyung Hee University, Seoul, Republic of Korea; Kyung Hee University, Seoul, Republic of Korea; Kyung Hee University, Seoul, Republic of Korea; Kyung Hee University, Seoul, Republic of Korea; Kyung Hee University, Seoul, Republic of Korea; Kyung Hee University, Seoul, Republic of Korea; Kyung Hee University, Seoul, Republic of Korea; Kyung Hee University, Seoul, Republic of Korea

## Abstract

Intrinsic capacity (IC) progressively decreases with age, accelerating the risk of disability. However, it is less known whether IC transition is associated with disability. We aimed to investigate the association between IC transition and incident disability among community-dwelling older adults. A retrospective analysis was conducted over a 6-year follow-up with biennial measurements, including 2,104 participants (52.7% women; mean age 76.1 ± 3.7 years) from the Korean Frailty and Aging Cohort Study. Participants were categorized based on the IC transition from the baseline to Wave 2: “Remained well and improved”, “Worsened”, and “Remained poor”. Disability was defined as a need for assistance in any items in activity of daily living. Incident disability was measured at Wave 3 and 4. Multivariate logistic regression was used to assess the relationship between IC transition and incident disability. The IC transition probability for “Remained well and improved” was the highest at 72.0%, followed by 15.8% for “Remained poor” and 12.2% for “Worsened”. Furthermore, over a 4-year follow-up, the incidence of disability among participants was 6.2% (n = 131). Compared with the “Remained well and improved”, the “Worsened” (odds ratio [OR]: 1.73, 95% confidence interval [CI]:1.01–2.98) and the “Remained poor” (OR: 2.34, 95% CI:1.45–3.79) had an increased risk of incident disability after adjusting covariates. Worsened IC and Remained poor groups were significantly associated with an increased risk of incident disability, compared to Remained well and improved group. Our findings suggest that early monitoring of IC is meaningful to prevent disability.

